# Versatility of the Supraclavicular Flap in Head and Neck Reconstruction

**Published:** 2020-06-05

**Authors:** F. Martins de Carvalho, Bernardo Correia, Álvaro Silva, Joana Costa

**Affiliations:** Plastic, Reconstructive and Aesthetic Surgery Department and Burn Unit, Centro Hospitalar de São João, Porto, Portugal

**Keywords:** supraclavicular artery perforator flap, head and neck reconstruction, pharyngoesophageal reconstruction, pharyngocutaneous fistula, tracheoesophageal fistula

## Abstract

**Objective:** Head and neck oncologic resections often leave intricate defects whose reconstruction remains a challenge. The pedicled supraclavicular artery perforator flap is an emerging option, and its applicability in head and neck reconstruction is gaining popularity. **Methods:** A retrospective analysis of patients regarding medical history, surgical indication, surgical technique, postoperative complications, and outcomes was carried out on all patients admitted to undergo pedicled supraclavicular artery perforator flap reconstruction within our institution. **Results:** Nine pedicled supraclavicular artery perforator flap reconstructions were performed. Surgical indications were 3 pharyngocutaneous fistulas, 2 tracheoesophageal fistulas, 2 cutaneous defects, 1 immediate pharyngoesophageal reconstruction, and 1 cutaneous and intraoral defect. All flaps survived completely. Excluding one patient who required a second flap due to plate reexposure, the remaining functional and esthetic outcomes were good. **Conclusions:** The pedicled supraclavicular artery perforator flap is reliable, is quick to harvest, and entails minimal donor site morbidity. Also, it is thin, is pliable, and has a wide arc of rotation, making it extremely versatile. It is an option that should be added to the spectrum of solutions of any head and neck reconstructive surgeon.

Head and neck oncologic resections often leave intricate defects whose reconstruction remains a challenge. The complex anatomy, the multiplicity of associated functions, and the desire for an esthetically favorable outcome all contribute to the inherent difficulty of these reconstructions. Advances in microsurgery often lead plastic surgeons to the top of the reconstructive ladder, avoiding morbid regional flaps. However, not all patients are candidates for microsurgery. The pedicled supraclavicular artery perforator (SAP) flap is an emerging option, and its applicability in head and neck reconstruction is gaining popularity.

The first vascular anatomical studies in the supraclavicular region were made by Mathes and Vasconez^1^ in the late **1070s** who presented the cervicohumeral flap. Further studies were carried out by Lamberty and colleagues,^2^^,^^3^ who, in 1983, named the supraclavicular artery. However, the applicability of this flap was controversial. Despite its axial pattern, the use of too long flaps led to reports of distal necrosis, reducing its popularity.^4^ This period also corresponded to the widespread use of concurrent flaps such as the pectoralis major or the trapezius.^5^ Pallua and colleagues^6^^,^^7^ rediscovered this flap in the 1990s, and the first report regarding its applicability to postburn mentosternal contractures was published in 1997, followed by oncologic head and neck reconstructions.

The purpose of this article is to demonstrate the indications, complications, and outcomes of the SAP flap reconstruction performed within our institution.

## METHODS

A retrospective analysis of the clinical charts and iconographic information of patients regarding medical history, surgical indication, surgical technique, postoperative complications, and outcomes was carried out on all patients admitted to undergo SAP flap reconstruction within our institution.

## RESULTS

### Medical history and surgical indications

Between July 2016 and December 2017, we performed 9 SAP flap reconstructions. Surgical indications were 3 pharyngocutaneous fistulas, 2 tracheoesophageal fistulas, 2 cutaneous defects, 1 immediate pharyngoesophageal reconstruction, and 1 cutaneous and intraoral defect (Table 1).

### Surgical technique

SAP flaps were designed with an axis drawn from the center of a triangle formed by the dorsal edge of the sternocleidomastoid muscle, the external jugular vein, and the medial part of the clavicle, toward the acromioclavicular joint and the ventral surface of the deltoid muscle. In this axis, the supraclavicular artery was consistently found using Dopper flowmetry before surgery and confirmed intraoperatively with a sterile probe. The flap was elevated from distal to proximal in a subfascial plane. The pedicle was never skeletonized and always included the superficial fascial system and the related platysma for approximately the width of the flap. The maximal length from the center of the described triangle was 20 cm. Flap widths longer than 7 cm required skin grafting for donor site closure. All flaps were harvested in about 45 minutes. They were then tunneled to the defect and the intervening tissue deepithelialized.

### Selected cases

#### Patient 2: Immediate pharyngoesophageal reconstruction

**Figure 1:**
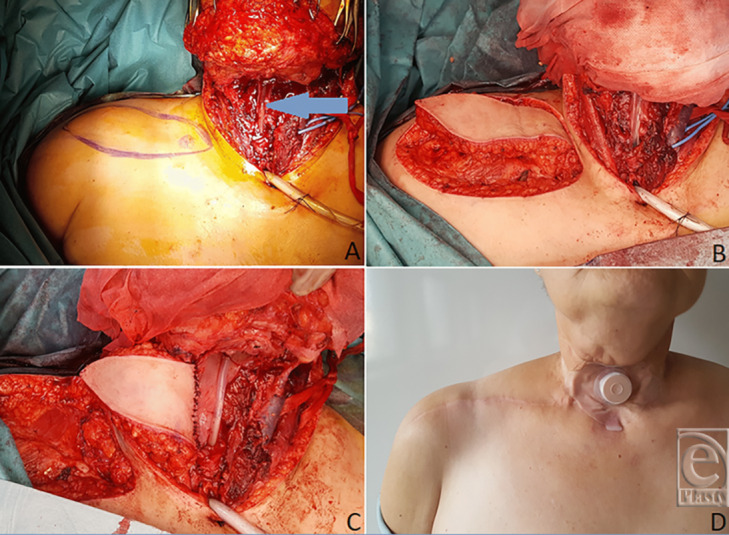
Case 2. (*a*) Posterior pharyngoesophageal wall with the nasogastric tube (blue arrow). (*b*) SAP flap raised. (*c*) SAP flap partially sutured to the posterior pharyngoesophageal wall. (*d*) Postoperative result at 16 months.

A 49-year-old woman with T_3_N_0_ laryngeal carcinoma underwent total laryngectomy along with lateral pharyngeal wall resection and bilateral nodal dissection levels II-IV. Only a narrow strip of about 7 cm in length of the posterior pharyngoesophageal wall remained. A tubularized SAP flap of 15 × 7 cm was raised for reconstruction. The postoperative period was uneventful. The patient started oral feeding on postoperative day 12, and no fistula occurred. Both the neck and donor sites were closed primarily. At 21-month follow-up and 17 months after completion of radiotherapy, there is no evidence of dysphagia, fistula, or stricture (Figs 1*a*-1*d*).

#### Patient 4: Tracheoesophageal fistula

**Figure 2:**
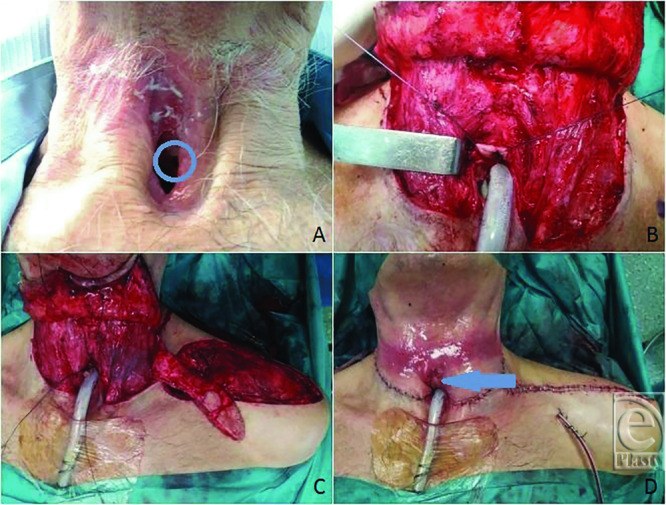
Case 4. (*a*) Tracheoesophageal fistula at the level of the tracheostoma (blue circle). (*b*) Anterior esophageal wall isolated. (*c*) SAP flap raised with 2 skin paddles. (*d*) Final aspect with the peristomal paddle visible (blue arrow).

A 68-year-old man with a history of total laryngectomy 6 years before presented with a pharyngocutaneous fistula at the level of the tracheostoma. He underwent reconstruction with an SAP flap with 2 skin paddles, one for the anterior esophageal wall and the other for the posterior tracheal wall, achieving a 2-layer closure. The donor site was closed primarily. Besides a donor site hematoma managed conservatively, the postoperative period was uneventful. The patient started oral feeding on postoperative day 14, and no fistula occurred. At 12-month follow-up, there is no evidence of dysphagia, fistula, or stricture (Figs 2*a*-2*d*).

#### Patient 6: Cutaneous and intraoral defect

**Figure 3:**
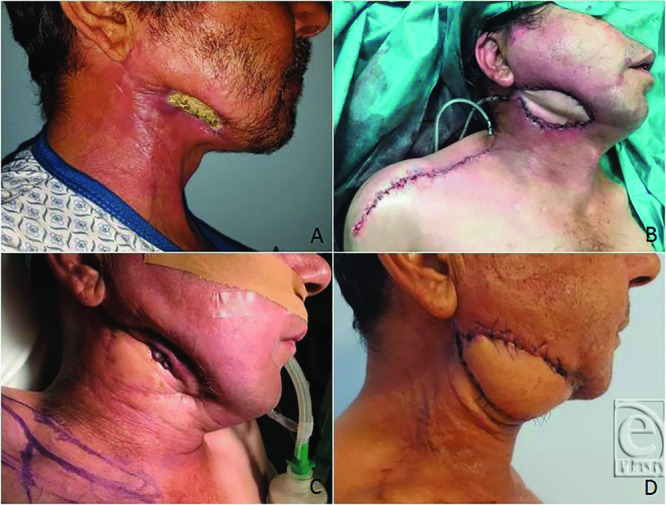
Case 6. (*a*) Osteoradionecrosis of the mandible with plate exposure. (*b*) Reconstruction with segmental mandibulectomy, reconstruction plate, and an SAP flap. (*c*) Exposure of the reconstruction plate through the SAP flap. (*d*) Coverage with a pectoralis major flap.

A 49-year-old man with osteoradionecrosis of the mandible with plate exposure and intraoral fistulization underwent segmental mandibulectomy and reconstruction with an SAP flap with 2 skin paddles and a reconstruction plate. The donor site was closed primarily. In the immediate postoperative period, the patient showed cellulitis and small dehiscence of the external paddle that healed with conservative measures. One month after discharge, the patient exhibited an ulceration and plate exposure through the SAP flap and underwent reconstruction with a pectoralis major flap. At 8-month follow-up, there is no evidence of tissue instability (Figs 3*a*-3*d*).

### Complications and outcomes

All flaps survived completely. Patients 3 and 5 had persistent leaking. Patient 3 required initiation of negative pressure wound therapy with fistula closure, and patient 5 was managed conservatively. Patient 6 had reexposure of the plate and required a second flap (see previously). Patients 1, 5, and 7 died at 16-, 5-, and 1-month follow-up, respectively, due to the underlying pathology.

Color match for cutaneous defects was very good. No donor site complications were found beyond hematoma managed conservatively and slight scar enlargement. No functional shoulder morbidity was reported.

## DISCUSSION

The reconstructive ladder in head and neck oncologic resections has largely shifted toward free tissue transfer because local flaps are often of inadequate size and regional flaps are often bulky and hard to inset in intricate defects and carry significant donor site morbidity. However, microsurgery is not without drawbacks. It requires time and expertise, and not all patients are candidates due to comorbidities or lack of recipient vessels.^8^^,^^9^

The growing knowledge of the angiosome of the supraclavicular artery led Pallua and colleagues^6^^,^^7^ to rediscover the SAP flap. Since then, it has been used in neck, tracheostomal, pharyngeal wall, mandible, parotid, and posterolateral skull base defects.^8^^,^^9^ In 1999, Liu and Chiu^10^ reported the first series of SAP flaps for partial or circumferential pharyngeal defects.

The supraclavicular artery branches from the transverse cervical artery (TCA) in a triangle formed by the external jugular vein, the sternocleidomastoid muscle, and the clavicle. In 90% of the cases, it arises from the middle third of the clavicle and in the remaining from the lateral third. After a short course perpendicular to the TCA, the supraclavicular artery turns toward the acromioclavicular joint and the ventral surface of the deltoid muscle. The mean distance between the origin of the supraclavicular artery and the sternoclavicular joint is on average 7.45 cm, and it has a mean course of 3.75 cm before piercing the deep fascia.^5^^,^^9^^,^^11^^,^^12^ When possible, the flap should be harvested on the right to avoid thoracic duct injury. Previous neck irradiation and previous or concurrent nodal dissection level IV/V are not contraindications to SAP flap harvest.^5^^,^^13^

Concerning flap dimensions, on a reported series of 103 SAP flaps, the authors suggest that a size of 22 × 10 cm can be elevated safely. Larger flaps may require skin grafting the donor site, and longer flaps should be supercharged.^11^ In our experience, flaps wider than 7 cm required skin grafting.

To increase the reach of the flap, it is possible to ligate the TCA distally to the supraclavicular artery origin. This way the pivot point of the flap becomes the origin of the TCA in the thyrocervical trunk.^11^ In our series, there was no need to perform this step. In all dissections, we did not skeletonize the vessels and preserved a fascial pedicle of approximately the width of the flap. This technique, described by Di Benedetto et al,^14^ ensures good reliability and protects the pedicle from tension or kinking. In addition, it makes flap harvesting easier and quicker.

Pallua and Wolter^15^ have recently described a variation of the original SAP flap, called the anterior supraclavicular artery perforator (a-SAP) flap. This flap should be designed toward the deltoideo-pectoral fossa and is perfused by the anterior supraclavicular artery that also originates from the TCA. This flap can be longer than the classic SAP flap reaching 35 cm and has the additional vantage of being even more thin.^15^

In our small series, the SAP flap proved to be very useful in pharyngoesophageal reconstruction. Three patients had pharyngocutaneous fistulas and 2 had tracheoesophageal fistulas. The latter underwent reconstruction with an SAP flap with 2 skin paddles in order to achieve a 2-layer closure. Patient 2 had a nearly complete circumferential defect and the reconstruction was uneventful. Other regional options, such as the internal mammary artery perforator, pectoralis major, or deltopectoral flaps, are often bulky, of insufficient size, or associated with more morbidity. Although muscle flaps atrophy, in pharyngeal reconstructions, it can lead to strictures. Besides that, muscle bulk can preclude primary neck closure and cause obstruction of the tracheostoma.^8^^,^^13^ Free tissue transfer (radial forearm, jejunal, and anterolateral thigh flaps) should be considered essentially backup options.^10^

One patient with pharyngocutaneous fistula (patient 3), prior radiation therapy, and 2 failed attempts of reconstruction with pectoralis major flap had persistent leaking that required initiation of negative pressure wound therapy with fistula closure after 2 months. Negative pressure wound therapy has been described as an effective treatment option in initial or recurrent pharyngocutaneous fistulas.^16^ We do not believe that leaking was due to partial flap necrosis because it healed without further surgical intervention, the flap was well perfused after inset, and native tissues were not healthy. Patient 5 had an expected leaking in the inferior border of the flap due to fistulization from the pyriform sinus. The achieved goal was to ameliorate the local conditions in order to support radiation therapy. Patient 6 had reexposure of the plate through the SAP flap. The thinness of the flap may make it less suitable for these reconstructions. However, the flap may have been placed under slight tension (Figs 3*a*-3*d*).

No donor site complications were found beyond hematoma and slight scar enlargement. No functional shoulder morbidity was reported. For cutaneous defects, the thickness, color, and texture match were very good. The SAP flap is also relatively hairless, which is beneficial in mucosal reconstructions. Excluding patient 6, who required a second flap, the remaining functional and esthetic outcomes were very good.

The SAP flap is reliable, is quick to harvest, has excellent color match to head and neck, and entails minimal donor site morbidity. Also, it is thin, is pliable, and has a wide arc of rotation from the skull base to esophagus, making it extremely versatile in small-volume tridimensional defects. It is an emerging alternative that should be added to the spectrum of solutions of any head and neck reconstructive surgeon.

## Figures and Tables

**Table 1:** Patient data*

Patient	Age, y	Background	Surgical indication	Defect size	Donor site	Previous radiotherapy	Previous reconstructive attempts	Hospitalization, d	Follow-up, mean, mo	Complications
1	62	T4 parotid carcinoma with auricular invasion	Cutaneous defect	11 × 7 cm	SG	No	No	9	16	None^†^
2	49	T3 laryngeal carcinoma	Pharyngoesophageal defect	Anterior and lateral pharyngoesophageal walls—7 × 6 cm	CP	No	No	17	21	None
3	54	Previous total laryngectomy	Pharyngocutaneous fistula	Anterior esophageal wall—4 × 1 cm	CP	Yes	Two pectoralis major flaps	30	12	Hematoma of the donor site Persistent fistula that required negative pressure wound therapy with fistula closure
4	68	Previous total laryngectomy	Tracheoesophageal fistula	Anterior esophageal and posterior tracheal walls—1.5 × 1.5 cm	CP	No	Sternocleidomastoid muscle flap	4	12	Hematoma of the donor site
5	47	T1 floor of the mouth carcinoma and T2 pyriform sinus carcinoma with cervical ulceration	Cutaneous defect	9 × 7 cm	SG	No	No	2	5	Persistent fistula^†^
6	49	Osteoradionecrosis of the mandible with plate exposure and intraoral fistulization	Cutaneous and intraoral defect	6 × 2 cm (cutaneous)—1 × 1 cm (intraoral)	CP	Yes	No	9	8	*Early*: Cellulitis and small dehiscence of the external paddle managed conservatively *Late*: Reexposure of the plate
7	75	Previous total laryngectomy	Pharyngocutaneous fistula/pharyngeal dehiscence	Anterior esophageal wall—6 × 2 cm	CP	No	No	42	2	None^†^
8	50	Previous total laryngectomy	Pharyngocutaneous fistula/pharyngeal dehiscence	Anterior esophageal wall—4 × 1 cm	CP	No	No	13	4	None
9	47	Previous total laryngectomy	Tracheoesophageal fistula	Anterior esophageal and posterior tracheal walls—2 × 1 cm	CP	Yes	Sternocleidomastoid muscle flap	5	4	None

*SG indicates skin graft; CP, closed primarily.

^†^Patients 1, 5, and 7 died at 16-, 5-, and 1-month follow-up, respectively, due to the underlying pathology.
